# Transcriptional Mutagenesis Induced by 8-Oxoguanine in Mammalian Cells

**DOI:** 10.1371/journal.pgen.1000577

**Published:** 2009-07-24

**Authors:** Damien Brégeon, Paul-Antoine Peignon, Alain Sarasin

**Affiliations:** Laboratoire Génomes et Cancer, CNRS FRE-2939, Institut Gustave Roussy, Villejuif, France; Stanford University School of Medicine, United States of America

## Abstract

Most of the somatic cells of adult metazoans, including mammals, do not undergo continuous cycles of replication. Instead, they are quiescent and devote most of their metabolic activity to gene expression. The mutagenic consequences of exposure to DNA–damaging agents are well documented, but less is known about the impact of DNA lesions on transcription. To investigate this impact, we developed a luciferase-based expression system. This system consists of two types of construct composed of a DNA template containing an 8-oxoguanine, paired either with a thymine or a cytosine, placed at defined positions along the transcribed strand of the reporter gene. Analyses of luciferase gene expression from the two types of construct showed that efficient but error-prone transcriptional bypass of 8-oxoguanine occurred *in vivo*, and that this lesion was not repaired by the transcription-coupled repair machinery in mammalian cells. The analysis of luciferase activity expressed from 8OG:T-containing constructs indicated that the magnitude of erroneous transcription events involving 8-oxoguanine depended on the sequence contexts surrounding the lesion. Additionally, sequencing of the transcript population expressed from these constructs showed that RNA polymerase II mostly inserted an adenine opposite to 8-oxoguanine. Analysis of luciferase expression from 8OG:C-containing constructs showed that the generated aberrant mRNAs led to the production of mutant proteins with the potential to induce a long-term phenotypical change. These findings reveal that erroneous transcription over DNA lesions may induce phenotypical changes with the potential to alter the fate of non-replicating cells.

## Author Summary

The DNA molecule is used as a template for duplication, to transmit genetic information to the progeny of a given cell, but also as a template for the transcription machinery. This machinery converts genetic information from the DNA form to the RNA form used for protein synthesis. Chemical alterations of the DNA molecule caused by endogenous or environmental stresses are responsible for the generation of mutations. Indeed, these lesions can induce replication errors when DNA is duplicated during cell division. These mutations have been shown to be responsible for many genetic diseases and other sporadic diseases, such as cancer. However, less is known about their effects on transcription. We report here that a specific DNA lesion may lead to erroneous transcription events, ultimately leading to the production of aberrant proteins. The magnitude of these errors seems to depend largely on the DNA sequences surrounding the lesion and the capacity of the cell to repair this lesion. We also show that the production of aberrant protein from the erroneous transcription products may affect the phenotype of the cells concerned. Lesion-induced transcription errors may also play a role in the development of neurodegenerative diseases, such as Alzheimer's and Parkinson's diseases.

## Introduction

During replication, DNA lesions exert deleterious effects by either blocking the DNA polymerase or allowing for mutagenic bypass of the lesion, which may be of major importance for evolution, hereditary diseases and cancer [1]. However, outside the unnatural environment of the laboratory, few cells undergo continuous cycles of division, and most cells exist instead in a non replicating state [2]. For example, several of the organs of multicellular organisms consist principally of non-dividing cells, the lifespan of which is limited by the functional differentiation associated with their normal physiology. These cells do not replicate their genome, but must nonetheless express a large number of genes for their physiological maintenance, which depends on the fidelity of both DNA transcription and mRNA translation. DNA lesions may be caused by a plethora of physical and chemical agents present in the natural environment. RNA polymerases would therefore be expected to encounter such lesions frequently, but much less is known about the interaction of the transcription machinery with such lesions than about the effects of these lesions on replication.

Most studies concerning the relationships between RNA polymerases and DNA lesions focus on bulky or distortive DNA damages. Such damage generally arrests elongation and is eliminated by transcription-coupled repair (TCR). This subpathway of the nucleotide excision repair pathway removes RNA polymerase II (RNApolII)-arresting lesions from the transcribed strand (TS) of genes by recruiting the DNA excision machinery [3]. However, some DNA lesions are bypassed by an elongating RNApolII *in vitro*, which can miscode at the lesion site and produce mutant transcripts with high efficiency via a process known as transcriptional mutagenesis (TM) [4],[5]. Interestingly, it was recently reported that even distortive DNA lesions, such as 8,5′-cyclo-2′-deoxyadenosine and cyclo-pyrimidine dimer, are bypassed at low frequency by human RNApolII *in vivo*, leading to the production of mutant transcripts [6]. If these events occur in cells, then each round of transcription of the sequence including the lesion would result in the production of an mRNA with a targeted change that will be translated multiple times to produce a relatively large population of mutant proteins. TM may therefore induce major phenotypical changes and important biological outcomes, particularly in cells that are not dividing [4],[7].

A frequently occurring DNA lesion results from the direct oxidation of guanine to generate 7,8-dihydro-8-oxoguanine (8OG) [8]. In *Escherichia coli*, 8OG is bypassed by the RNA polymerase, leading to TM events due to the insertion of adenine or no nucleotide opposite to this lesion [9]. Several *in vitro* studies have indicated that 8OG could also be the source of TM in human cells, as it does not represent a strong block for an elongating RNA polII and, in various experimental conditions, the bypass of this lesion has been shown to result in the erroneous incorporation of adenine opposite to the lesion [10]–[12]. The tendency of 8OG to induce TM in murine cells was also reported in a recent study [13]. In this study, we focused on the outcome of 8OG-mediated TM in mammalian cells, including human cells in particular.

A *Photinus pyralis* luciferase (*Pp*luc) reporter system has been used to examine the occurrence of 8OG-mediated TM in diverse mammalian cells and to investigate the effects of DNA repair capacity on these TM events in human and mouse cells. Two types of construct were used in this study, in which 8OG was introduced into the transcribed strand of the *Pp*luc gene, opposite either a thymine (8OG:T) or a cytosine (8OG:C). In cells, the 8OG:T mispair constitutes a poor substrate for DNA repair mechanisms. Consequently, many rounds of transcription over 8OG occur before the complete removal of this lesion from the transcribed strand of this type of construct [14],[15]. This would result in an amplification of 8OG-induced TM events, thereby facilitating studies of such events. However, most of guanine oxidation process leads to 8OG:C pairs in DNA, which are rapidly processed through hOGG1-mediated base excision repair (BER) [14]. We therefore also investigated the ability of 8OG-mediated TM events to induce a transient phenotypical change with 8OG:C-containing constructs.

An analysis of the relative *Pp*luc activity expressed from 8OG:T mispair-containing constructs showed that the extent of 8OG-mediated TM is, similarly to what has been found for DNA polymerase, largely dependent on the context sequence and, probably on the relative distance of the lesion from the promoter. *Pp*luc mRNAs expressed from 8OG:T mispair-containing constructs were extracted from cells with high levels of TM and sequenced, to identify the spectrum of RNApolII misinsertion events induced by transcription over the 8OG lesion. Quantification of the *Pp*luc activities expressed from 8OG:C-containing constructs confirmed the hypothesis that the extent of TM depends strongly on the DNA repair capacity of the cell. Such quantification also showed that TM was a potential source of a long-term phenotypical change, even in cells with a normal DNA repair background. With both types of construct, we assessed the effect of the level of reporter gene expression on TM by modifying the amount of reporter mRNA produced, using a dose-dependent doxycycline-responsive promoter. Furthermore, the *Pp*luc activities expressed from both types of construct in various TCR-deficient cells provided insight into the role of this mechanism in the repair of an 8OG lesion in the transcribed strand of a gene. These observations may have potentially important implications for the etiology of diseases, including those affecting non-dividing cells in particular.

## Results

### A reporter system for TM

**Figure 1:**
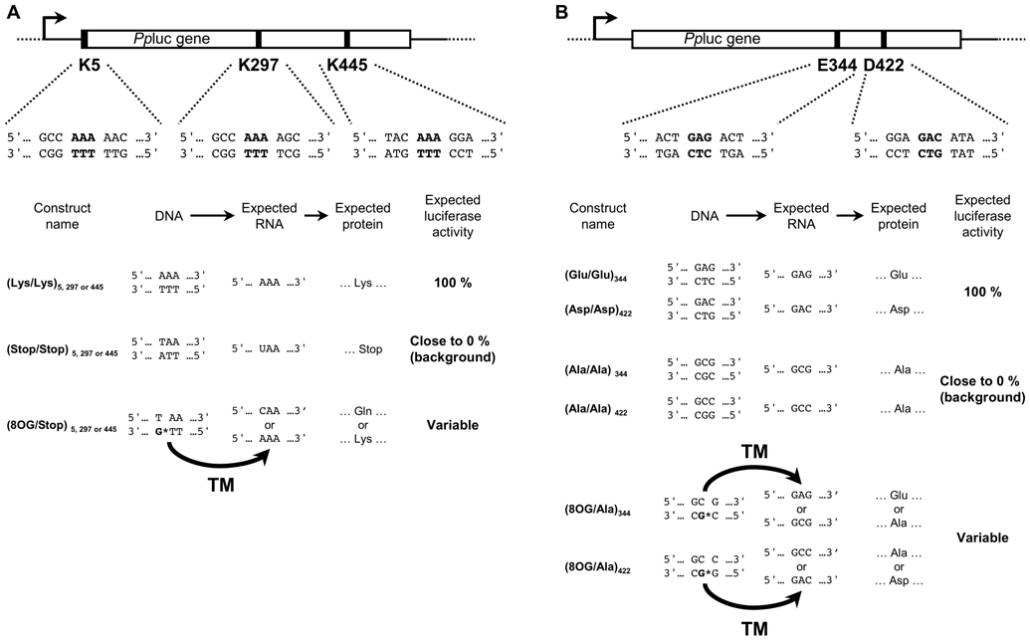
*Pp*luc gene modification inducing transcriptional mutagenesis and phenotypical change in mammalian cells. Five codons of the *Pp*luc gene were modified, to investigate the effects of the presence of an 8OG residue in the transcribed strand (TS) of a gene. (A) Codons 5, 297 and 445 were modified to specify lysine (K) (LFS/Lys), a stop codon (LFS/Stop) or lysine through transcriptional mutagenesis (TM) and glutamine (Gln) through faithful transcription (8OG/Stop). (B) Codon 344 was modified to encode glutamic acid (LFS/Glu)_344_ or alanine (LFS/Ala)_344_ leading to the production of an active or an inactive E344A *Pp*luc, respectively. Codon 422 was modified to specify either aspartic acid (LFS/Asp)_422_ or alanine (LFS/Ala)_422_, leading to the production of an active or an inactive D422A *Pp*luc, respectively. In (8OG/Ala) constructs, transcriptional bypass of the 8OG leads to the production of active *Pp*luc through TM or the production of inactive *Pp*luc through faithful transcription.

We investigated the effect of 8OG on transcription and phenotypical change in mammalian cells by using a reporter assay to measure the levels of active *Pp*luc generated from expression constructs derived from the pBDA6 plasmid (Figure S1) and containing DNA lesions at defined positions on the TS of the gene (Figure 1). Five sets of three constructs were generated with the following nomenclature (lesion-free strand (LFS) or 8OG-containing strand (8OG) / amino acid specified on the NTS)_codon number_ and composed as follows: (i) a wild-type construct with the wild-type sequence of the *Pp*luc gene [(LFS/Lys)_5_, (LFS/Lys)_297_, (LFS/Glu)_344_, (LFS/Asp)_422_ and (LFS/Lys)_445_]; (ii) an 8OG-containing construct [(8OG/Stop)_5_, (8OG/Stop)_297_, (8OG/Ala)_344_, (8OG/Ala)_422_ and (8OG/Stop)_445_] in which the 8OG was introduced into the TS of the specified codon and (iii) a mutant construct [(LFS/Stop)_5_, (LFS/Stop)_297_, (LFS/Ala)_344_, (LFS/Ala)_422_ and (LFS/Stop)_445_] (Figure 1). In three of the mutant constructs, a lysine codon within the *Pp*luc gene (codon number 5, 297 or 445) was replaced by a premature stop codon, resulting in the production of an inactive C-terminally truncated protein [16]. The other two mutant constructs, specifying alanine at codon 344 or 422, resulted in the production of an inactive form, E334A or D422A, of the *Pp*luc protein (Branchini, B.R. personal communication). In transfected cells, expression of the *Pp*luc gene from these different constructs was driven by the dose-dependent doxycycline-responsive *P*_tight_ promoter and protein activity was normalized with respect to the *Renilla reniformis* luciferase (*Rr*luc). Both luciferases are independently translated from the same polycistronic mRNA, with *Rr*luc translation initiated at an internal ribosome entry site (IRES) located between the two open reading frames (Figure S1).

For each assay and for each cell line (Table 1), normalized *Pp*luc activities measured after transfection with wild-type constructs was set as the 100% reference point for quantifying relative *Pp*luc activities expressed from the same cell line transfected with mutant or 8OG-containing constructs. The relative *Pp*luc activity of the cell lines transfected with mutant constructs was very low and varied from 0.001% to 0.022% (Table 2 and Table 3). These results confirm that the method used to generate the constructs was appropriate for this study and that expression of the *Pp*luc gene from mutant constructs resulted in the production of inactive proteins. The 10^4^– to 10^5^–fold difference in *Pp*luc activity between wild-type and mutant constructs is large enough for measurement of the intermediate levels of activity potentially generated by the TM events induced by 8OG.

**Table 1:** Cell lines used in this study.

Cell line	Complementation group/phenotype	Source	Reference
MRC5V1	Normal	C. Arlett	[48]
VA13	Normal	P.C. Hanawalt	[49]
CS3BE-S3G1	CS-A	A. Sarasin	[50]
CS1AN-SV	CS-B	C. Arlett	[50]
XPCS2BA-SV	XP-B/CS	W.J. Kleijer^a^	[51]
XP-CS2-SV	XP-D/CS	A. Sarasin	[52]
XPCS1LV-SV	XP-G/CS	Corriel^a^	[53]
HCT116	MLH1	F. Praz	[54]
DLD-1	MSH6	F. Praz	[55]
LoVo	MSH2	F. Praz	[56]
MEF	Normal	S. Boiteux	[17]
MEF *ogg1 ^−/−^*	Null mOgg1	S. Boiteux	[17]

a Primary cell strains were immortalized, in our laboratory, by transfection with the pLAS-wt plasmid carrying the TAg from SV40, as previously described [44].

**Table 2:** Relative luciferase activity 24 h after transfection of the various cell lines with constructs with lesions at codons 5, 297 and 445.

Constructs (LFS/Stop)		NTS 5′ … TAA … 3′^a^	
		TS 3′ … ATT … 5′	
Cell line	Codon 5	Codon 297	Codon 445
MRC5V1	0.002^b^	0.022	0.004

Constructs (LFS/Gln)		NTS 5′ … CAA … 3′^a^	
		TS 3′ … GTT … 5′	
Cell line	Codon 5	Codon 297	Codon 445
MRC5V1	315^b^	63.5	4.97

Constructs (8OG/Stop)		NTS 5′ … T AA … 3′^a^	
		TS 3′ … G*TT … 5′	
Cell line	Codon 5	Codon 297	Codon 445
MRC5V1	0.52^b^	21.44	51.26
VA13	0.38	16.45	50.99
CS3BE-S3G1	0.21	12.33	45.26
CS1AN-SV	0.19	16.43	43.06
XPCS2BA-SV	0.91	15.71	49.45
XP-CS2-SV	0.88	9.97	54.56
XPCS1LV-SV	0.22	11.65	46.26
HCT116	0.25	15.94	38.00
DLD-1	0.41	14.25	20.76^c^^,^ d
LoVo	0.79	38.5	72.43^d^^,^ e
MEF	0.15	8.21	56.00
MEF ogg1 ^−/−^	0.76^d^^, ^ f	22.61^d^^, ^ f	59.73
MRC5V1 (low)^g^	0.10	17.26	41.52

a NTS: nontranscribed strand; TS: transcribed strand ; G*: 8OG.

b Values are expressed as [(RLU*_Pp_*/RLU*_Rr_*)_construct_/(RLU*_Pp_*/RLU*_Rr_*)_100%_]×100 in the same cell line. Each value is the mean of at least six replicate samples. RLU: relative light units.

c p values for the Mann-Whitney U test are 0.0374 and 0.0250 for comparisons with the relative luciferase activities of MRC5V1 or VA13 cells transfected with the same construct, respectively.

d Distributions were considered to be significantly different when p<0.05.

e Both p values for the Mann-Whitney U test are <0.0001 for comparisons with the relative luciferase activities of MRC5V1 or VA13 cells transfected with the same construct.

f p values for Mann-Whitney U test are <0.0001 and 0.0022 for comparisons with the relative luciferase activities of MEF cells transfected with (8OG/Stop)_5_ and (8OG/Stop)_297_, respectively.

g The dose of doxycycline was reduced from 2 µg/ml to 1 ng/ml.

**Table 3:** Relative luciferase activity 24 h after the transfection of various cell lines with constructs with lesions at codons 344 and 422.

	Constructs	
	NTS 5′ … GCG … 3′^a^	NTS 5′ … GCC … 3′^a^
Cell line	TS 3′ … CGC … 5′	TS 3′ … CGG … 5′
MRC5V1	0.005^b^	0.001
VA13	0.006	0.001
MEF	0.002	0.004

	Constructs	
	NTS 5′ … GC G … 3′^a^	NTS 5′ … GC C … 3′^a^
Cell line	TS 3′ … CG*C … 5′	TS 3′ … CG*G … 5′
MRC5V1	0.76^b^	0.28
VA13	2.13	1.25
CS3BE-S3G1	0.30	0.16
CS1AN-SV	1.00	0.75
XPCS2BA-SV	0.65	0.31
XP-CS2-SV	0.43	0.29
XPCS1LV-SV	0.74	0.27
MEF	0.41	0.23
MEF ogg1 ^−/−^	14.94^c^^, ^ d	17.09^c^^, ^ d
MRC5V1 (low)^e^	0.38	0.18

a NTS: nontranscribed strand; TS: transcribed strand ; G*: 8OG.

b Values are expressed as [(RLU*_Pp_*/RLU*_Rr_*)_construct_/(RLU*_Pp_*/RLU*_Rr_*)_100%_]×100 in the same cell line. Each value is the mean of at least six replicate samples. RLU: relative light units.

c p values for Mann-Whitney U test are 0.0022 for comparisons with the relative luciferase activities of MEF cells transfected with the same construct.

d Distributions were considered to be significantly different when p<0.05.

e The dose of doxycycline was reduced from 2 µg/ml to 1 ng/ml.

### 8OG:T-driven TM depends on the sequence context

**Figure 2:**
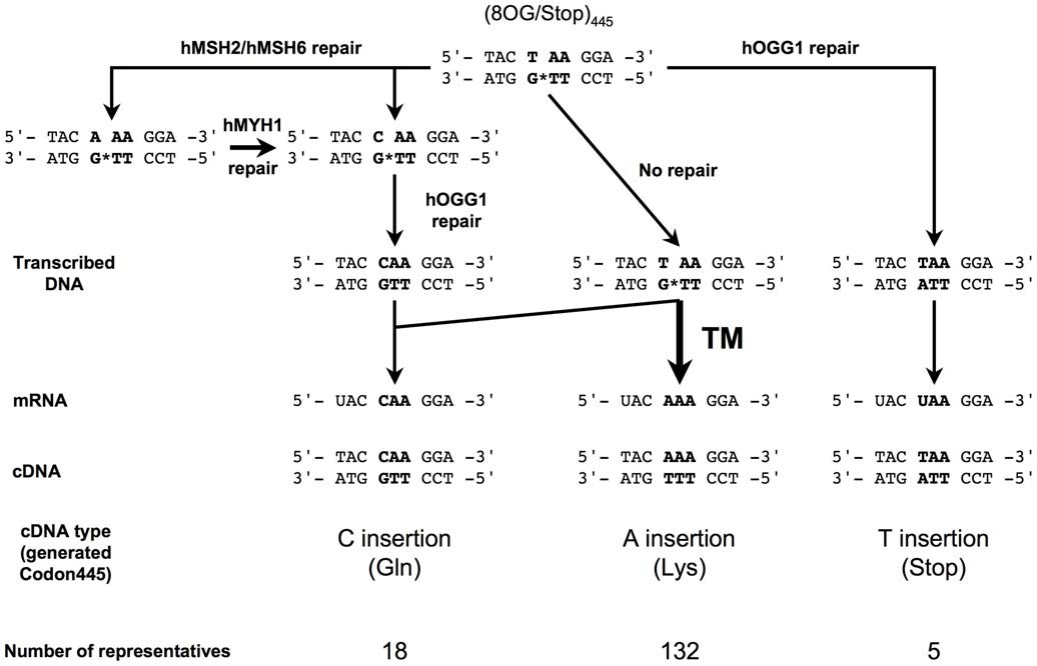
8OG-driven transcriptional mutagenesis in human cells. We provide here a schematic diagram of the fate of an 8OG:T mispair at codon 445. After transfection with (8OG/Stop)_445_, the DNA molecule can be repaired by various DNA repair pathways (see text for details). Depending on the pathway, transcription of the repaired molecules leads to the production of an mRNA molecule containing either a C or a U as the first base of codon 445, thus generating a stop codon or encoding a glutamine, respectively. The transcription of unrepaired molecules is the source of 8OG-driven TM events. We assessed the frequency of such events by extracting total RNA from MRC5V1 cells transfected with the (8OG/Stop)_445_ construct 24 hours after transfection. A portion of the *Pp*luc mRNA was amplified and RT-PCR fragments were subcloned into pUC18 for sequencing. The numbers of each type of cDNA are indicated for each type of base insertion.

The extent of 8OG-induced TM was determined with (8OG/Stop) constructs, which contain an 8OG:T mispair in codon 5, 297 or 445 (Figure 1). Transcription through the lesion and the insertion of adenine or cytosine opposite to the 8OG would result in a *Pp*luc mRNA encoding lysine or glutamine at the corresponding codon. The insertion of a lysine residue at this position results in fully active wild-type *Pp*luc, whereas the insertion of a glutamine residue at position 5, 297 or 445 leads to the production of a *Pp*luc protein with activity levels 5% to 315% that of the wild-type *Pp*luc (Table 2). Alternatively, base excision repair (BER) of this 8OG would result in the production of a *Pp*luc mRNA containing a premature stop codon, which would therefore not give rise to an active *Pp*luc (Figure 2).

*Pp*luc activities were 100 to 1,000 times higher in normal cells transfected with (8OG/Stop) constructs than in normal cells transfected with (LFS/Stop) constructs. The relative *Pp*luc activities expressed from (LFS/Stop) constructs were very low and similar in all cell lines tested, whereas the relative activity of *Pp*luc measured in normal human (MRC5V1 and VA13) and murine (MEF) cells transfected with (8OG/Stop) constructs depended strongly on the position of the lesion in the TS of the *Pp*luc gene (Table 2). These relative activities are indeed ranging from less than 1%, if the 8OG was located at codon 5, to more than 50% if the 8OG was located at codon 445 (Table 2). In cells transfected with (LFS/Stop) constructs, activities of the coexpressed *Rr*luc were systematically high and similar to those measured in cells transfected with wild-type constructs, thus ruling out the involvement of nonsense-mediated decay in the modulation of relative *Pp*luc activity, because the same transcript encodes both luciferases. The repair of 8OG:T mispairs in normal cells therefore seems to depend largely on the sequence context and, possibly, on the distance between the promoter of the transcribed gene and the mispair.

### TM does not depend on expression level

Differential 8OG:T mispair repair in a transcribed gene could potentially be affected by the level of expression of the gene. We tested this hypothesis by lowering the level of *Pp*luc/*Rr*luc mRNA production by decreasing the amount of doxycycline in the recovery medium for transfected cells (Table 2). A comparison of *Rr*luc activities shows that production of the reporter mRNA under control of the pTight promoter decreased by a factor of about 10 when the concentration of doxycycline wasdecreased from 2 µg/ml to 1 ng/ml (data not shown). The relative *Pp*luc activities expressed in MRC5V1 cells transfected with (8OG/Stop) constructs were similar for both doxycycline concentrations. Similar results were also obtained with 8OG:C-containing constructs (Table 3). Taken together, these results indicate that, over the range tested, the expression level of the mRNA does not affect the 8OG repair process and that 8OG-induced TM events occur at similar frequency whether the gene is strongly or weakly expressed.

### MMR and BER are involved in 8OG:T mispair repair

The *Pp*luc relative activity expressed in cells transfected with (8OG/Stop) constructs is directly correlated with the efficiency of 8OG:T mispair repair. It has been shown *in vitro* that 8OG can be removed from an 8OG:T mispair-containing DNA molecule by either hOGG1-driven BER or by the mismatch repair system (MMR), in an hMSH2/hMSH6-dependent manner [14],[15].

The role of OGG1-driven BER in the differential 8OG:T repair efficiency was deciphered by quantifying the relative *Pp*luc activity expressed from normal (MEF) or *Ogg1*-deficient (MEF *ogg1 ^−/−^*) murine cell lines transfected with (8OG/Stop) constructs [17]. No significant difference in relative *Pp*luc activity was observed between MEF and MEF *ogg1 ^−/−^* cells transfected with the (8OG/Stop)_445_ construct, whereas the relative *Pp*luc activities of MEF *ogg1 ^−/−^* cells transfected with (8OG/Stop)_5_ or (8OG/Stop)_297_ were significantly higher by factors of 5 and 2.7, respectively, than those of MEF cells transfected with the same constructs (Table 2). The impact of MMR on the differential repair efficiency of an 8OG:T mispair was assessed by using our constructs to transfect hMLH1- (HCT116), hMSH6- (DLD-1) or hMSH2-deficient (LoVo) cells. Relative *Pp*luc activities expressed in hMSH2-deficient cells transfected with (8OG/Stop) constructs were higher than those in normal cells, whereas the relative activities of hMSH6- and hMLH1-deficient cells were lower than those in normal cells (Table 2). The only significant differences with respect to normal cells (MRC5 and VA13) were obtained for hMSH2- and hMSH6-deficient cells transfected with the (8OG/Stop)_445_ construct, indicating a possible key role of these proteins in the 8OG MMR-dependent repair pathway. These results indicate that both MMR and BER are involved in repairing 8OG:T mispairs *in vivo*.

### Base-specificity of 8OG-driven TM

The high relative *Pp*luc activities expressed in cells transfected with the (8OG/Stop)_445_ construct and the consistently high levels of *Rr*luc activity in cells transfected with (8OG/Stop), in which *Rr*luc activity levels were similar to those in cells transfected with wild-type constructs, suggest that the presence of an 8OG on the TS of a gene does not block transcription and that the human and murine RNApolII enzymes incorporate adenine or cytosine opposite to 8OG. However, detectable enzyme activity cannot be viewed as direct evidence for TM, as both nucleotide insertions result in the production of *Pp*luc enzymes with various degrees of activity.

As mentioned above, the 8OG:T mispair in the (8OG/Stop)_445_ construct constitutes a very poor substrate for DNA repair and many rounds of transcription occur before the removal of the lesion from the DNA template. Thus, analyses of *Pp*luc mRNA sequences produced in cells transfected with the (8OG/Stop)_445_ construct should provide an accurate description of the spectrum of misinsertion events occurring during transcription over an 8OG lesion. We identified the nucleotides inserted opposite 8OG by the human RNApolII by sequencing partial cDNA subclones obtained from RNA extracted from MRC5V1 cells transfected with the (8OG/Stop)_445_ construct. The major cDNA type (85%) harbors an AAA lysine codon at position 445, the expected sequence when adenine is incorporated opposite to 8OG through TM (Figure 1 and Figure 2). The other two minor cDNA types contain a TAA stop codon (3%), reflecting the transcription of repaired (8OG/Stop)_445_ molecules, or a CAA glutamine codon (12%). Thus, in human cells, RNApolII can generate mutated transcripts containing an adenine residue at the position corresponding to the lesion during transcription over 8OG.

### 8OG:C-driven TM induces phenotypical change

The use of (8OG/Stop) constructs provided important insight into the repair of an 8OG:T mispair in cells and the spectrum of nucleotide insertions occurring opposite to the 8OG lesion during *in vivo* transcription by human RNApolII over this lesion. However, 8OG:T mispairs occur only rarely *in vivo*, because guanine oxidation mostly generates 8OG:C pairs. The ability of 8OG to induce a phenotypical change through TM was investigated with (8OG/Ala) constructs containing an 8OG:C pair at codon 344 or 422 (Figure 1). Active *Pp*luc proteins can be produced from these constructs only through the insertion of an adenine residue opposite to the 8OG, resulting in the production of an mRNA with the wild-type *Pp*luc gene sequence. Although 8OG:C pair is a good substrate for OGG1-mediated repair, levels of relative *Pp*luc activity in human (MRC5V1 and VA13) and murine (MEF) cell lines transfected with (8OG/Ala) constructs were from 57- to 1300-fold higher than those obtained following transfection of these same cell types with (LFS/Ala) constructs (Table 3). Thus, *in vivo*, the murine and human RNApolII enzymes can transcribe through an 8OG lesion, inducing the misincorporation of adenine opposite to this lesion, resulting in a significant phenotypical change.

### BER can modulate 8OG-driven phenotypical change, whereas TCR cannot

The magnitude of this phenotypical change may depend on the DNA repair capacity of the cells, as repair of the 8OG would convert codon 344 or 422 to an alanine codon, leading to the production of inactive *Pp*luc. We assessed the extent to which the phenotypical change depended on the DNA repair capacity of the cells by transfecting mouse cells lacking OGG1-mediated BER with (8OG/Ala) constructs [17]. The relative *Pp*luc activities of MEF *ogg1 ^−/−^* cells transfected with (8OG/Ala)_344_ or (8OG/Ala)_422_ were 36.4- and 74.3-fold higher, respectively, than those for the normal parental cell line (MEF) transfected with the same constructs (Table 3). These findings thus demonstrate that the impact of TM on the phenotype depends on the DNA repair capacity of the cells (Table 3).

An 8OG lesion in a TS might also be repaired by pathways other than OGG1-mediated BER, possibly including TCR, as cells from patients with Cockayne syndrome have been shown to be defective for both TCR and the repair of oxidative lesions [18]. Nonetheless, the role of TCR in the repair of oxidative lesions, such as 8OG, remains debatable, as several papers addressing this question have recently been retracted [19]–[21]. In our system, the TCR-mediated repair of 8OG should be revealed by a higher level of phenotypical change in TCR-deficient cells transfected with (8OG/Ala) constructs and higher relative *Pp*luc activities in cells transfected with (8OG/Stop) constructs. However, the relative *Pp*luc activities expressed from CS- and XP/CS-derived cells transfected with (8OG/Ala) or (8OG/Stop) constructs fell within the same range as those for normal cells transfected with these constructs (Table 2 and Table 3), ruling out the possibility of TCR-mediated repair of 8OG.

### Evolution in the 8OG-driven phenotypical change over time

The change in phenotype observed for normal cells transfected with (8OG/Ala) should not be permanent, as the 8OG lesion responsible for inducing this change should be repaired over time. We evaluated the magnitude of the phenotypical change induced by 8OG over time by assessing the production of active *Pp*luc at various times after the transfection of MRC5V1 cells.

**Figure 3:**
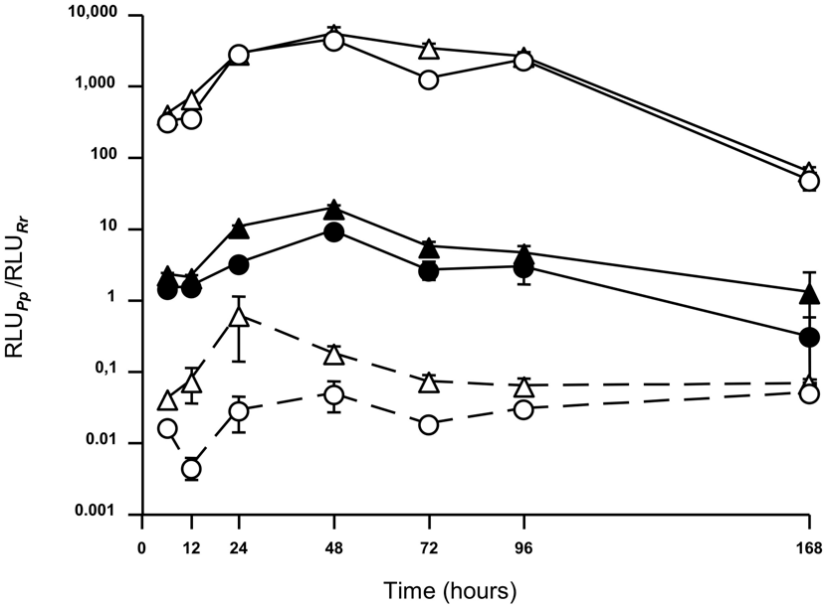
The pattern of 8OG-driven phenotypical change over time. MRC5 cells were transfected with (LFS/Glu)_344_ (open triangles, solid lines), (LFS/Asp)_422_ (open circles, solid lines), (8OG/Ala)_344_ (closed triangles, solid lines), (8OG/Ala)_422_ (closed circles, solid lines), (LFS/Ala)_344_ (open triangles, dashed lines) or (LFS/Ala)_422_ (open circles, dashed lines). *Pp*luc and *Rr*luc activities in transfected cells were quantified at different time points after transfection at time 0. Each experimental point corresponds to the mean of six replicates±the standard error of the mean. RLU: relative light units.

Higher levels of active *Pp*luc were consistently expressed from (8OG/Ala) constructs than from (LFS/Ala) constructs, over a period of at least seven days after transfection (Figure 3). The observed differences were significant for up to four days after transfection, but the difference observed on day 7 was not significant as, for each construct, only one of the six replicates displayed levels of *Pp*luc activity above the background, a phenomenon similar to the so-called “mutagenesis jackpot” [22]. Similar decreases in *Pp*luc activities were observed with wild-type and (8OG/Ala) constructs, but these results clearly indicate that the TM process induced by 8OG can lead to a long-term phenotypical change in the affected cells.

## Discussion

In the present study, we investigated the *in vivo* consequences of the presence of an 8OG moiety in the transcribed strand (TS) of the *Pp*luc reporter gene in human and murine cells. Enzyme activity measurements and mRNA sequence analysis results showed that transcription over the 8OG on the template strand (TS) generated mutated transcripts, leading to a long-term phenotypical change. Furthermore, the magnitude of the observed phenotypical change depended strongly on the DNA repair capacity of the cells, but not on the level of expression of the gene.

### 8OG-mediated TM in human cells

*In vitro* studies have shown that 8OG does not block the progression of the mammalian RNApolII and that non mutagenic cytosine insertions opposite to this lesion are favored, although the insertion of a certain number of adenine residues is also detected [10]–[12],[23]. Analysis of the cDNA population generated from the *Pp*luc mRNA produced in MRC5V1 cells transfected with the (8OG/Stop)_445_ construct revealed that in vivo transcription of 8OG generates two distinct populations of transcripts. The largest of these two populations consisted of mutated mRNA molecules containing an adenine residue incorporated opposite to the 8OG during transcription. The other population consisted of transcripts in which a cytosine residue was incorporated at the position corresponding to the lesion, probably due to non mutagenic transcription over 8OG. This type of cDNA could potentially result from faithful transcription over across the 8OG lesion, but may also result from the transcription of (8OG/Stop)_445_ molecules repaired by MMR. Indeed, it has been shown that the binding of hMSH2/hMSH6 to an 8OG:T mispair can promote excision of the 8OG-free strand and that adenine and cytosine are inserted with similar efficiencies opposite to the 8OG during repair synthesis, resulting in 8OG:A- or 8OG:C-containing molecules [15]. For 8OG:A, a two-step pathway has been proposed in which the incorporated adenine is excised by hMYH and a cytosine is inserted during repair synthesis [1],[24]. The resulting 8OG:C-containing DNA is then used as a substrate for hOGG1 [25], which can replace the 8OG by a guanine residue, creating a glutamine codon (3′-GTT-5′) in the TS of the *Pp*luc gene (Figure 2). Saxowsky *et al.* recently reported cytosine incorporation to be the major event observed during 8OG bypass by murine RNApolII, with adenine incorporation observed in about 10% of transcripts [13]. This apparent discrepancy may be due to differences in sequence context. As reported above, our results clearly indicate that sequence context may have a major influence on the outcome of 8OG-induced TM in mammalian cells. The nature of the nucleotide paired with the 8OG in the DNA template may also account for this difference. Indeed, if 8OG is placed opposite a cytosine residue, about 28% of the transcripts contain an adenine at the position corresponding to the lesion after the expression of their reporter gene in MEF *ogg1 ^−/−^* cells [13]. Our findings are consistent with those of Saxowsky *et al.*, because we found that 15 to 20% adenine-containing transcripts were produced when 8OG:C pair-containing constructs were expressed in MEF *ogg1 ^−/−^* cells (Table 3). Therefore, these results indicate that, *in vivo*, adenine insertion by human RNApolII during transcription across an 8OG lesion is a major event. Our findings clearly demonstrate that TM can be induced during transcription over an 8OG lesion *in vivo*. Consequently, 8OG must be removed from the DNA before RNApolII encounters this lesion, to avoid the production of mutant transcripts and mutant proteins. Significant DNA repair pathway activity is therefore required in conditions of non-growth in the absence of DNA replication.

### TM–induced phenotypical change and evolution over time

**Figure 4:**
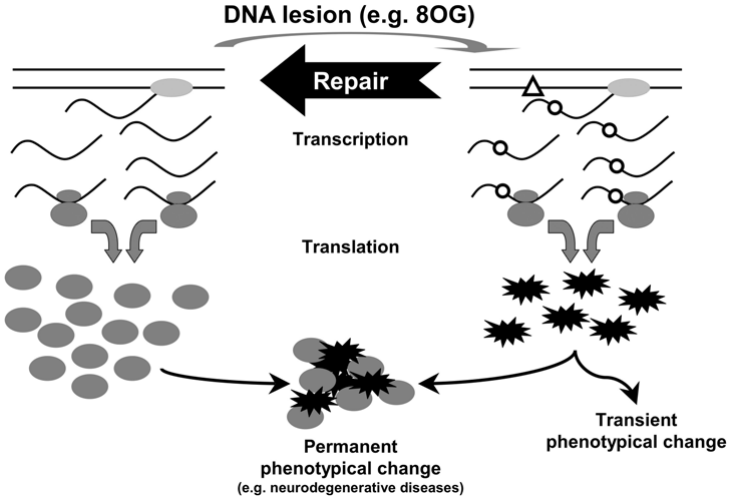
Speculative biological outcomes of TM. In normal conditions (left part), the expression of a gene in non-dividing cells results in the production of normal proteins. When a DNA lesion (e.g. 8OG) occurs in the transcribed strand of a gene (on the right), multiple RNA polymerase bypasses of the lesion result in misincorporation events (e.g. A instead of C opposite to 8OG, open circle on the mRNA) at the same position in most of the mRNA molecules produced before the DNA is repaired. A large population of mutated mRNA molecules can then be translated multiple times to generate large amounts of mutated protein, which may induce a transient phenotypical change. However, if the mutated proteins are resistant to protein degradation and have a dominant effect, the phenotypical change may be prolonged or even permanent. For example, the mutated proteins may be more likely to form aggregates, providing a nucleation point for the recruitment of normal proteins produced after the DNA lesion is repaired. These protein aggregates may therefore mimic a dominant-negative effect ultimately resulting in cell degeneration, as observed in neurodegenerative diseases.

The use of an 8OG:T mispair-containing construct was crucial for analysis of the specificity of base incorporation opposite to this lesion during transcription by RNApolII. This mispair probably occurs rarely in cells, because guanine oxidation in DNA mostly results in the production of 8OG:C pairs, the best substrate for OGG1-mediated repair [26]. However, even in cells not deficient for any of the known DNA repair pathways, significant amounts of active *Pp*luc protein were expressed in cells transfected with 8OG:C pair-containing constructs. Thus, in mammalian cells, the oxidation of a guanine residue in the TS of a gene may lead to major phenotypical changes, as the only difference between the (LFS/Ala) and (8OG/Ala) constructs is the replacement of a normal guanine residue by 8OG (Figure 1). This simple and only difference allows for cells transfected with (8OG/Ala) constructs to express non negligible amounts of active *Pp*luc protein through TM, rendering them phenotypically different from the same cells transfected with (LFS/Ala) constructs, which produce no active *Pp*luc protein. Thus, when there is an 8OG residue present in the TS of an expressed gene, RNApolII continually produces transcripts containing a G to A base substitution at the same position, potentially leading to a phenotypical change. The long-term consequences of this phenotypical change for the cell depend on the time required to repair the lesion inducing them and, particularly, the half-life of the mutant protein produced (Figure 4). Unexpectedly, we continued to detect active *Pp*luc protein (the “mutant” form in this case) for up to seven days after transfection with (8OG/Ala) constructs. Knowing that *Pp*luc is not a very stable protein, as its half-life was estimated to be of no more than four hours in mammalian cells [27], TM must therefore continue for a prolonged period of time in human cells. This finding has important implications concerning the role of TM in the etiology of diseases, particularly those affecting non-dividing cell populations, caused by the generation of mutant proteins by TM.

### Phenotypical change is modulated by BER, but not by TCR

In mammalian cells, the main pathway for the removal of 8OG from DNA involves the OGG1 glycosylase protein. It has been shown that *ogg1^−/−^* mice accumulate 8OG lesions in their DNA with aging, leading to a moderate tissue-specific increase in spontaneous mutation rate; these findings demonstrate the antimutator role of the OGG1 BER pathway [17],[28]. The relative activity of *Pp*luc expressed in MEF *ogg1 ^−/−^* cells transfected with (8OG/Ala) constructs reflects the higher level of mutant transcript production in these cells, leading to a more pronounced phenotypical change than observed in the normal parental cells. This implies that a deficiency or decrease in the activity of this enzyme, as observed in several diseases [29],[30], may induce a phenotypical change in some cells of the body, contributing to the etiology of the disease.

We also investigated the role of TCR in the repair of 8OG lesions in the TS *in vivo*. The role of this process in removing non bulky oxidative lesions, such as 8OG, from the TS is, as aforementioned, quite controversial [19]–[21]. It is thought that repair events of this type involve the blockage of RNApolII elongation by a lesion on the TS, providing a signal for the recruitment of the TCR machinery. In this regard, the role of TCR in the removal of 8OG from a TS has been investigated in several studies focusing on RNApolII interactions at sites containing this lesion [10]–[12],[23]. These studies concluded that, both *in vitro* and *in vivo*, 8OG only weakly blocks elongation by the mammalian RNApolII. In this study, the two luciferases were generated by independent translation from the same polycistronic mRNA, with the translation of *Rr*luc initiated at an IRES located between the two open reading frames. A blockage of RNA polII elongation during the transcription of this mRNA would thus result in very weak *Rr*luc luminescence. However, the observation that cells transfected with 8OG-containing or wild-type constructs had similar levels of *Rr*luc protein activity strongly suggests that, *in vivo*, 8OG does not represent a strong block to an elongating mammalian RNApolII. Furthermore, in the five TCR-deficient human cell lines obtained from patients with Cockayne syndrome or XP/CS, the relative *Pp*luc activities resulting from the expression of (8OG/Ala) constructs were not significantly different from those in cells with normal DNA repair capacities. This suggests that the 8OG lesion in these constructs was repaired equally efficiently in TCR-deficient and normal cells. These results represent direct evidence that TCR does not play an important role in the repair of 8OG lesions in human cells, consistent with the most recent results obtained *in vitro* [11],[12],[31]. Furthermore, Saxowsky *et al.* also reported that 8OG was repaired equally efficiently in murine TCR-deficient and normal cells [13]. These independent *in vivo* observations are clarifying a controversial area of the DNA repair field.

The relative activity of *Pp*luc expressed in MEF *ogg1 ^−/−^* cells transfected with (8OG/Ala) constructs suggests that other DNA repair activities may also be involved in the repair of 8OG:C pairs in cells. The activities involved may include that of hNTH1, as this BER N-glycosylase has been shown to cleave duplex oligonucleotides containing 8OG [32]. Alternatively, 8OG may be removed from the DNA by glycosylases of the NEIL family [33],[34].

### Expression level has no impact on the magnitude of TM

Accessibility to 8OG may influence the efficiency of lesion repair and, consequently, the magnitude of 8OG-mediated TM events. In our system, the reporter gene is under the control of a dose-dependent doxycycline-responsive promoter, facilitating the modulation of expression levels. The relative activity of *Pp*luc expressed in MRC5V1 cells cultured with low doses of doxycycline were similar to that obtained in the presence of high doses of the transcription inducer. This observation reveals that expression level and thus accessibility to the lesion does not play a major role in the modulation of TM-mediated events occurring in cells.

### Insights from the TM induced by an 8OG:T mispair

It has frequently been reported that 8OG:T mispairs may be processed by both OGG1-mediated repair and MSH2/MSH6-dependent MMR pathways [15],[26]. Nonetheless, the difference in affinity of the OGG1 protein and of the MSH2/MSH6 complex for an 8OG:T mispair suggests that mispairs of this type are most likely to be processed in an MSH2/MSH6-dependent manner [15]. The relative activity of *Pp*luc expressed in OGG1-deficient or MMR-deficient cells transfected with 8OG:T-containing constructs suggests that both pathways play a role in the repair of this type of mispair in mammalian cells. However, the efficiency of 8OG:T mispair repair depends on its location within the transcribed gene. It therefore seems likely that the recognition of this mispair, by MSH2/MSH6 or OGG1, and the efficiency of its removal by MMR or BER may depend strongly on sequence context. We cannot rule out the possibility that the efficiency of these mechanisms also depends on transcription factors, as the efficiency of 8OG:T repair seemed to be correlated with the distance of this mispair from the transcriptional initiation sequence of the gene, creating a polarity gradient. A similar polarity gradient phenomenon has been reported during meiotic gene conversion in fungi. Indeed, the non reciprocal transfer of information from one chromatid to another during yeast meiosis often varies linearly from one end of the studied gene to the other (for review see [35]). This phenomenon was shown to be initiated from promoter-containing regions of the chromosome and to be dependent upon MMR. It remains unclear whether the observed polarity gradient along a transcribed reporter gene is a general feature of DNA repair mechanisms or due exclusively to the specific sequence context at codons 5, 297 and 445 of the *Pp*luc gene. It would also be interesting to use this reporter system to investigate whether the great variability of 8OG-induced TM is correlated with similar levels of variability in DNA polymerase errors during replication.

### Speculation on biological outcomes of TM

The potential outcomes of TM include a number of deleterious events initiated by mutant proteins, such as cell death and changes in cellular physiology [5],[7]. An “error catastrophe” scenario [36], in which age-related cell death may result from the corruption of genes required for normal cellular function and viability, may result from the accumulation of TM-generated mutant proteins. Indeed, age-dependent deficiencies in the import of OGG1 into the nuclear and mitochondrial compartments results in the accumulation of oxidative lesions, such as 8OG, which may lead to an age-related increase in the production of mutant or misfolded proteins [37]. Furthermore, some neurological disorders are characterized by aggregates of misfolded and aberrant proteins associated with an increase in DNA oxidation [38], mainly due to a decrease in hOGG1 activity in neuronal cells, resulting in the accumulation of large amounts of 8OG in the genomes [39]. These aggregates are very resistant to cellular degradation [40] and have a dominant-negative effect on cell survival. Indeed, the addition of aggregated proteins to the culture medium of human neuroblastoma cells is sufficient to induce apoptotic cell death [41], because these aggregates act as nucleation points for the normal protein [42]. Additionally, it has also recently been suggested that hypomorphic alleles of *hOGG1* are associated with Alzheimer's disease cases and that defects in OGG1 may play an important role in the disease in a significant number of AD patients [43]. Thus, as depicted in Figure 4, aberrant proteins with a dominant-negative effect produced through TM-related events may play an important role in the pathogenesis of neurodegenerative diseases, such as Alzheimer's disease and Parkinson's disease.

## Materials and Methods

### Cell lines and culture conditions

The cell lines used in this study are described in Table 1. They were cultured at 37°C, under an atmosphere containing 5% CO_2_, in minimal essential medium (MEM) supplemented with 10% fetal calf serum, 2 mM L-glutamine, 0.3% amphotericin B (Fungizone), 100 IU penicillin and 100 µg/ml streptomycin. XPCS2BA and XPCS1LV diploid fibroblasts were transformed, in our laboratory, with the pLAS-wt-plasmid carrying the TAg of SV40 [44].

### The luciferase reporter system

The pBDA6 luciferase reporter vector (Figure S1) contains the *Photinus pyralis* (firefly) and the *Renilla reniformis* (sea pansy) luciferase genes (*Pp*luc and *Rr*luc, respectively) organized in a bicistronic operon. *Rr*luc gene translation is initiated from the IRES located between the *Pp*luc and *Rr*luc open reading frames, and both luciferase proteins are thus translated from the same mRNA. Transcription to generate this polycistronic mRNA is initiated from the dose-dependent doxycycline-responsive *P*_tight_ promoter. When cells are transfected with this plasmid, the presence of doxycycline in the culture medium allows the transcriptional activator (rtTA) to bind the *P*_tight_ promoter, leading to the production of both luciferases. This plasmid also contains the ampicillin resistance gene (Amp^R^) and an origin of double-strand DNA replication (ColE1), allowing its propagation in bacterial cells. The production of circular single-stranded DNA corresponding to the coding strand of the *Pp*luc gene is initiated from the f1 origin of replication (f1 ori). The other elements of this plasmid are two SV40-polyadenylation sites (SV40pA) and an intervening sequence (IVS) directing the correct processing and stabilization of the mRNA in mammalian cells, and a *P*_CMV_ promoter for expression of the rtTA gene. This vector is deprived of mammalian origin of replication, to prevent artifacts generated by mutagenic replication of the 8OG-containing constructs.

The pBDA6 vector was constructed in several steps (Figure S2). First, nucleotides 6060 to 2614 from pIRES (Clontech Laboratories) and nucleotides 79 to 4360 from pTet-On (Clontech Laboratories) were amplified by PCR, using the *Pfu* Turbo DNA polymerase (Stratagene). The oligonucleotide primers used for these reactions were designed to create *Age*I and *Pac*I sites at either end of the amplified fragments. The two PCR products were then digested with *Age*I and *Pac*I (New England Biolabs) and ligated together, using T4 DNA ligase (Roche), to generate the pBDA5/1 plasmid. Nucleotides 4336 to 5084 of the pBDA5/1 plasmid were replaced by nucleotides 2590 to 343 from pTRE-Tight, resulting in the replacement of the *P*_CMV_ promoter by the *P*_Tight_ dose-dependent doxycycline-responsive promoter (Clontech Laboratories) and generation of the pBDA5/2 plasmid. The pBDA6 final construct was obtained by amplifying the *Pp*luc and *Rr*luc genes from pBI-Luc (Clontech Laboratories) and pRL-CMV (Promega), respectively. The *Pp*luc fragment was inserted upstream from the IRES, between the *Nsi*I and *Nhe*I sites, whereas the *Rr*luc fragment was inserted into the *Not*I site downstream from the IRES. All the variants (pBDA6-luc K5X, K5Q, K297X, K297Q, E344A, D422, K445X and K445Q), differing from the original by a single point mutation in the *Pp*luc gene, were obtained by directed mutagenesis, through the PCR of overlapping extensions technique [45]. The fragments generated were then digested with *Nhe*I and *Nsi*I and inserted into pBDA6 digested with the same enzymes. All PCR amplifications were performed using the *Pfu* Turbo DNA polymerase (Stratagene). The name of the pBDA6 indicates the change of amino-acid sequence of the *Pp*luc protein at the specified codon. The *Rr*luc and *Pp*luc genes of all the plasmids were sequenced by Genome Express (Meylan, France). All plasmid constructs were introduced into the DH12S strain of *Escherichia coli*. Bacteria were grown in LB supplemented with ampicillin (100 µg/ml) (Sigma).

### Template construction

We produced eighteen constructs: (LFS/Lys)_5_, (8OG/Stop)_5_, (LFS/Stop)_5_, (LFS/Gln)_5_, (LFS/Lys)_297_, (8OG/Stop)_297_, (LFS/Stop)_297_, (LFS/Gln)_297_, (LFS/Glu)_344_, (8OG/Ala)_344_, (LFS/Ala)_344_, (LFS/Asp)_422_, (8OG/Ala)_422_, (LFS/Ala)_422_,(LFS/Lys)_445_, (8OG/Stop)_445_, (LFS/Stop)_445_ and (LFS/Gln)_445_. The first part of the name of each construct indicates the strand transcribed: lesion-free strand (LFS) or 8OG-containing strand (8OG). The second part of the name indicates the amino acid specified by the non-transcribed strand and the single strand DNA of the pBDA6 variant used as a template for DNA synthesis. The index number corresponds to the codon number in the *Pp*luc gene. Single-stranded DNA was prepared, and templates constructed, as previously described [46],[47]. The primers used to initiate DNA polymerization reactions for the template construction are listed in Table S1.

### Luciferase activity measurement

Cells were transfected with constructs by nucleofection methods, using the NHDF nucleofector kit (Amaxa). Cells were first treated with trypsin and washed twice in 1×PBS (Gibco). For each transfection, 300,000 cells (or 1,000,0000 cells for normal MEF and LoVo cells) were resuspended in 100 µl of NHDF solution and mixed with 300 ng (or 1 µg for normal MEF cells) of the construct concerned. The mixture was then subjected to electroporation program U23 of the Amaxa nucleofector device. Immediately after the electric shock, cells were resuspended in 3 ml of MEM (Gibco) supplemented with 10% fetal calf serum (Gibco), 2 mM L-glutamine (Gibco) and 2 µg/ml (or 1 ng/ml when specified) doxycycline (Sigma) and placed in 6-well plates in an incubator maintained at 37°C, under an atmosphere containing 5% CO_2_.

The medium was removed from each well 24 hours after transfection, and cells were washed twice with cold 1×PBS. Cells were lysed by incubation for 45 minutes in 500 µl of Passive Lysis Buffer (Promega), placed at −20°C for 30 minutes and then thawed to room temperature. Luciferase activity was measured with the Dual-Luciferase Reporter Assay System (Promega), using 80 µl of “Luciferase Assay Reagent”, 80 µl of lysed cells and 80 µl of “Stop and Glo” reagent. Luminescence, in relative light units (RLU), was determined over a 10-second period, in a Femtomaster FB12 luminometer (Zylux Corp.). *Pp*luc activity was normalized with respect to *Rr*luc activity for each transfection, using the following formula: (RLUPp/RLURr). For each set of transfections with the same cell line, the relative *Pp*luc activity of cells transfected with 8OG-containing (or LFS/Stop or LFS/Ala) constructs was calculated as follows: [(RLU*_Pp_*/RLU*_Rr_*)_construct_/(RLU*_Pp_*/RLU*_Rr_*)_100%_]×100 with the 100% being the normalized *Pp*luc activity in cells transfected with the corresponding wild-type construct (LFS/Lys for codon 4, 297 and 445, LFS/Glu for codon 344 and LFS/Asp for codon 422).

### RNA extraction and RT–PCR

RNA was extracted from MRC5V1 cells 24 hours after transfection with the (8OG/Stop)_445_ construct. RNA was extracted with Tri-Reagent solution (Sigma), according to the manufacturer's instructions. Contaminating DNA was eliminated from the RNA solution by two treatments with the DNA-free kit (Ambion). We then used about 50 ng of RNA for RT-PCR with Superscript II (Invitrogen) as a reverse transcriptase and *Taq* DNA polymerase for amplification (New England Biolabs), using LBRT1 and LBRT2 as primers [9]. For each RNA preparation, the absence of DNA contamination was checked by amplification reactions in the same conditions but with the omission of the reverse transcriptase. The cDNA was subcloned by ligating a *Sau*3A/*Hin*cII fragment of the RT-PCR product between the *Bam*HI and *Hin*cII sites of pUC18 (all restriction enzymes were from New England Biolabs). Subclones were then amplified with Clo18L and Clo18U [9]. The DNA amplified from the subclones was sequenced by Genome Express (Meylan, France).

## Supporting Information

Figure S1 Plasmid used for the assessment of transcriptional mutagenesis in mammalian cells. The pBDA6 plasmid, the construction of which is shown in Figure S2, contains the following features: pTight (dose-dependent doxycycline-responsive promoter), IVS (intervening sequence), *Pp*luc (*Photinus pyralis* luciferase gene), IRES (internal ribosome entry site), *Rr*luc (*Renilla reniformis* luciferase gene), SV40pA (SV40 polyadenylation site), f1 ori (origin of single-stranded DNA replication), PCMV (CMV promoter), rtTA (reverse tetracycline-controlled transactivator), ColE1 (bacterial origin of double-stranded DNA replication), AmpR (beta-lactamase gene). The pBDA6 plasmid contains no mammalian origin of replication, so the presence of active *Pp*luc protein in transfected cells cannot be due to mutagenic replication of the 8OG-containing constructs in cells.
(0.09 MB TIF)

Figure S2 Stages in the construction of pBDA6. See text for details.
(0.12 MB TIF)

Table S1 Primers used in this study.
(0.03 MB DOC)
