# Supplementary figures and images for: Transcriptional Mutagenesis Induced by 8-Oxoguanine in Mammalian Cells
Source: PLoS Genet. 2009 Jul 24;5(7):e1000577. doi: 10.1371/journal.pgen.1000577 (PMC2708909; doi:10.1371/journal.pgen.1000577)

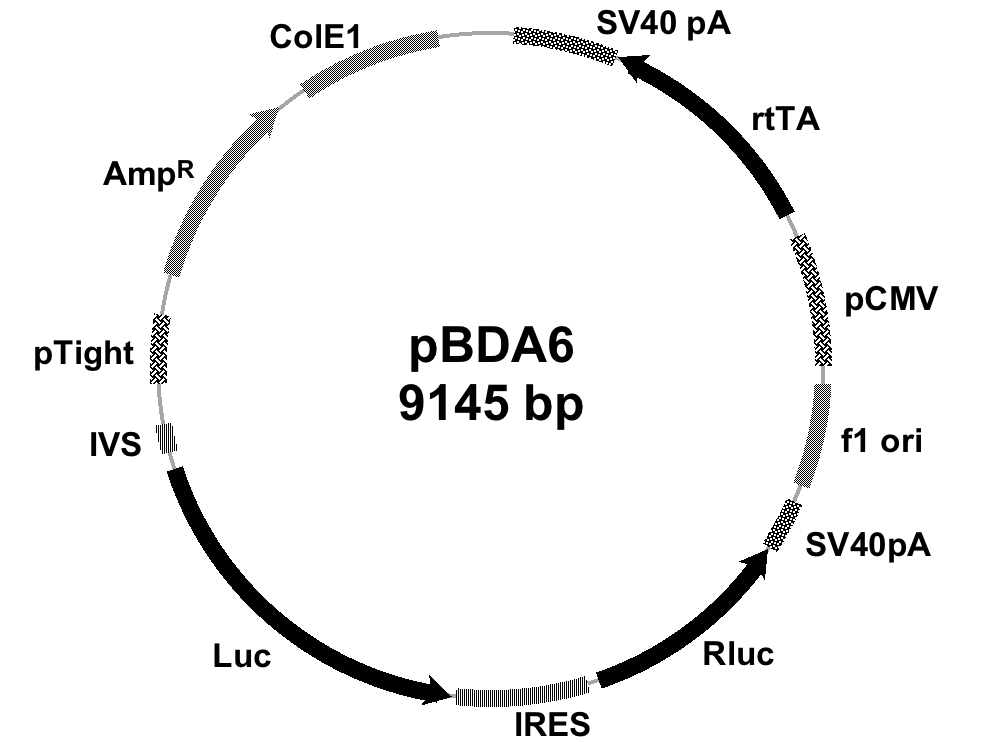

Supplement: Figure S1 — Plasmid used for the assessment of transcriptional mutagenesis in mammalian cells. The pBDA6 plasmid, the construction of which is shown in Figure S2, contains the following features: pTight (dose-dependent doxycycline-responsive promoter), IVS (intervening sequence), Ppluc (Photinus pyralis luciferase gene), IRES (internal ribosome entry site), Rrluc (Renilla reniformis luciferase gene), SV40pA (SV40 polyadenylation site), f1 ori (origin of single-stranded DNA replication), PCMV (CMV promoter), rtTA (reverse tetracycline-controlled transactivator), ColE1 (bacterial origin of double-stranded DNA replication), AmpR (beta-lactamase gene). The pBDA6 plasmid contains no mammalian origin of replication, so the presence of active Ppluc protein in transfected cells cannot be due to mutagenic replication of the 8OG-containing constructs in cells. (0.09 MB TIF) [file pgen.1000577.s001.tif]

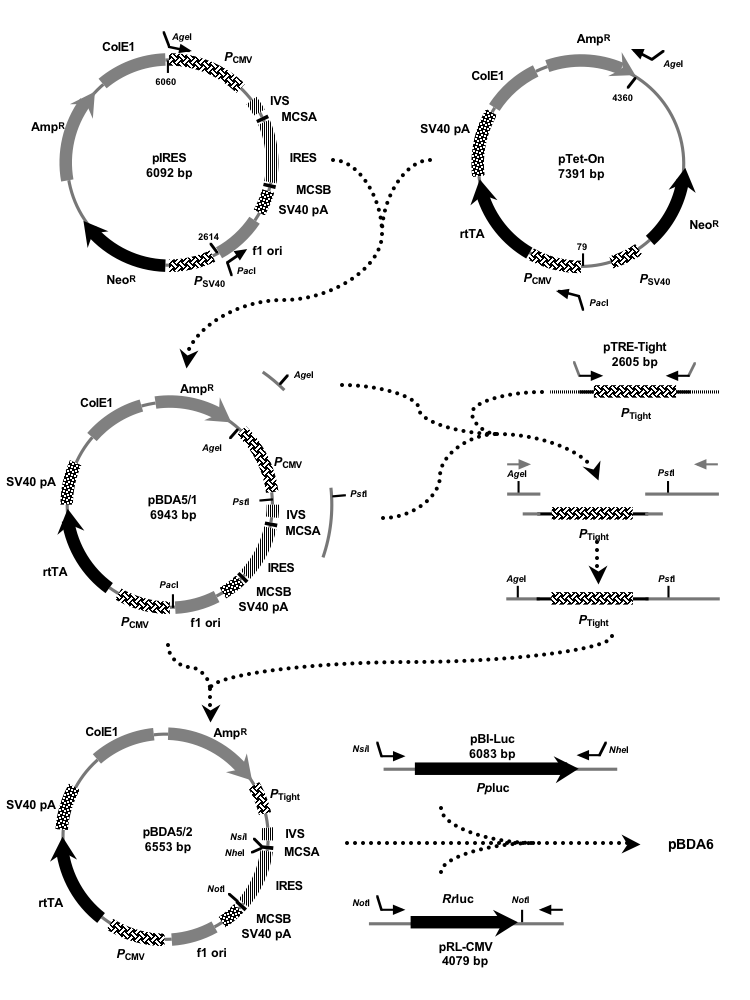

Supplement: Figure S2 — Stages in the construction of pBDA6. See text for details. (0.12 MB TIF) [file pgen.1000577.s002.tif]
