# Supplementary material for: Transcriptional Mutagenesis Induced by 8-Oxoguanine in Mammalian Cells
Source: PLoS Genet. 2009 Jul 24;5(7):e1000577. doi: 10.1371/journal.pgen.1000577 (PMC2708909; doi:10.1371/journal.pgen.1000577)
Supplement: Table S1 — Primers used in this study. (0.03 MB DOC) [file pgen.1000577.s003.doc]

| **Table S1: Primers used in this study** | |
| --- | --- |
| Primers | Sequence |
| 5-Lys  5-Stop  5-8OG  297-Lys  297-Stop  297-8OG  344-Glu  344-Ala  344-8OG  422-Asp  422-Ala  422-8OG  445-Lys  445-Stop  445-8OG | 5'phos-CGCCGGGCCTTTCTTTATGTTTT**T**GGCGTCTT^a^  5'phos-CGCCGGGCCTTTCTTTATGTTTT**A**GGCGTCTT^a^  5'phos-CGCCGGGCCTTTCTTTATGTTTT**G***GGCGTCTT^b^  5’phos-GTGCTTT**T**GGCGAAGAATGAAAATAGGGTTG^a^  5’phos-GTGCTTT**A**GGCGAAGAATGAAAATAGGGTTG^a^  5’phos-GTGCTTT**G***GGCGAAGAATGAAAATAGGGTTG^b^  5’phos-GGTGTAATCAGAATAGCTGATGTAGTC**T**CAGTGAGC^a^  5’phos-GGTGTAATCAGAATAGCTGATGTAGTC**G**CAGTGAGC^a^  5’phos-GGTGTAATCAGAATAGCTGATGTAGTC**G***CAGTGAGC^b^  5’phos-TCGTCTTCGTCCCAGTAAGCTATG**T**CTCCAGA^a^  5’phos-TCGTCTTCGTCCCAGTAAGCTATG**G**CTCCAGA^a^  5’phos-TCGTCTTCGTCCCAGTAAGCTATG**G***CTCCAGA^b^  5’phos-CTGGTATCCTT**T**GTATTTAATTAAAGACTTCAAGC^a^  5’phos-CTGGTATCCTT**A**GTATTTAATTAAAGACTTCAAGC^a^  5’phos-CTGGTATCCTT**G***GTATTTAATTAAAGACTTCAAGC^b^ |

^a^ Oligonucleotides purchased from Proligo France (Paris, France)

^b^ Oligonucleotides purchased from Operon (Köln, Germany)
